# Ministers of Health: short-term tenure for long-term goals?

**DOI:** 10.1590/S1516-31802011000200005

**Published:** 2011-03-03

**Authors:** Marcos Bosi Ferraz, Rafael Teixeira Azevedo

**Affiliations:** I MD, PhD. Professor and Director, Centro Paulista de Economia da Saúde (CPES), Universidade Federal de São Paulo (Unifesp), São Paulo, Brazil.; II Undergraduate student at the School of Medicine, Universidade Federal de São Paulo (Unifesp), São Paulo, Brazil.

**Keywords:** Leadership, Policy making, Health status indicators, Health policy, Decision making, Liderança, Formulação de políticas, Indicadores básicos de saúde, Política de saúde, Tomada de decisões

## Abstract

**CONTEXT AND OBJECTIVE:**

Healthcare investments should consider short and long-term demands. The objectives here were to compare the average tenures of ministers of health in Brazil and in another 22 countries and to evaluate the relationship between ministers' tenures and a number of indicators.

**DESIGN AND SETTING:**

Descriptive study conducted at Centro Paulista de Economia da Saúde (CPES).

**METHODS:**

Twenty-two countries with the highest Human Development Indices (HDIs) and Brazil were included. The number of ministers over the past 20 years was investigated through each country's Ministry of Health website. Pearson's correlation coefficient was used to compare the number of ministers in each country with that country's indicators. The Mann-Whitney test was used to compare ministers' tenures in Brazil and other countries.

**RESULTS:**

The mean tenure (standard deviation, SD) of Brazilian ministers of health was 15 (12) months, a period that is statistically significantly shorter than the mean tenure of 33 (18) months in the other 22 countries (P < 0.05). There was a moderate and statistically significant positive correlation between the number of ministers and mortality rates for several conditions. The number of ministers also presented moderate and statistically significant negative correlations with *per capita* total healthcare expenditure (r = −0.567) and with *per capita* government healthcare expenditure (r = −0.530).

**CONCLUSION:**

On average, ministers of health have extremely short tenures. There is an urgent need to think and plan healthcare systems from a long-term perspective.

## Abstract (pt)

**CONTEXTO E OBJETIVO:**

Investimentos em saúde deveriam considerar as demandas de curto e longo prazo. Os objetivos foram comparar o tempo médio no cargo dos ministros da saúde no Brasil e em outros 22 países e avaliar a relação entre o tempo médio dos ministros no cargo e alguns indicadores.

**TIPO DE ESTUDO E LOCAL:**

Estudo descritivo realizado no Centro Paulista de Economia da Saúde (CPES).

**MÉTODOS:**

Vinte e dois países com os Índices de Desenvolvimento Humano (IDHs) mais altos e o Brasil foram incluídos. O número de ministros da saúde nos últimos 20 anos foi pesquisado na página eletrônica do Ministério da Saúde de cada país. Coeficiente de correlação de Pearson (CCP) foi utilizado para comparar o número de ministros em cada país com os indicadores daquele país. O teste de Mann-Whitney foi usado para comparar o tempo de permanência do ministro da saúde no Brasil com os outros países.

**RESULTADOS:**

O tempo médio (desvio padrão, DP) no cargo dos ministros brasileiros foi de 15 (12) meses, um período estatisticamente significante menor do que a média observada nos outros 22 países, 33 (18) meses (P < 0,05). Foi observada uma moderada e também estatisticamente significante correlação positiva entre o número de ministros e taxas de mortalidade para várias condições. Houve uma moderada e significante correlação negativa entre o número de ministros e o investimento em saúde *per capita* total (r = −0.567) e o investimento em saúde público *per capita* (r = −0.530).

**CONCLUSÃO:**

O ministro da saúde permanece, na média, pouco tempo no cargo. Há uma premente necessidade de se pensar e planejar o sistema de saúde para o longo prazo.

## INTRODUCTION

Ideally, healthcare investment decisions should consider both short and long-term demands. As nations become more developed, with well structured healthcare systems, qualified resources and an educated population, it becomes harder to justify healthcare policy decisions that focus only on the short term. In less developed nations, the demand to alleviate the suffering of its already diseased population poses a tremendous challenge to its leaders in charge of healthcare policy and decision-making. Although preventive measures are obviously recognized and preferable to treatment measures, it is not possible to close our eyes to current demand and look only at the future.

Healthcare systems in many developing nations face a major challenge: how to meet 21^st^ century healthcare standards and the technology it demands with funds that, as a percentage of gross domestic product (GDP), are less than what developed nations were investing in healthcare in the 1980s. Furthermore, how can developing nations meet such expectations when they are still struggling with healthcare problems that rich countries overcame 40 or 50 years ago?^1^ This scenario stresses the importance of having long-term goals, while at the same time addressing short and medium-term needs, and recognizing the intrinsic limitations of the system itself.

It is entirely possible to use the concepts and methods of evidence-based medicine to demonstrate the effectiveness and quality of certain products and services. However, decisions on whether or not to provide them to the population require an accurate assessment of people's healthcare needs and expectations, as well as a system of priorities and an objective understanding of the structural limitations of the healthcare system itself. In other words, although there are very good products and services available, it may not be possible to deliver them to all developing nations in any general manner. The art of making well grounded and justified decisions should therefore avoid the type of decisions that would place a Ferrari (a high-quality product) off-road (a system lacking in infrastructure).^2^ Most, if not all healthcare policies in developing nations should bear this in mind.

Politics is a means of distributing power and strategy is a means of implementing policy.^3^ National policies typically reflect the composition of both the executive and the legislature.^4^

The Minister of Health, or a similarly named position in some countries, is usually the chief executive officer (CEO) of the healthcare system. Although there is significant variation between countries, the Ministry of Health is supposed to provide overall strategic direction and leadership for the healthcare system as well as helping to develop legislation, regulations and policies to support the strategic directions. Nowadays, it is equally important to face the challenge of and need for innovation within the healthcare system, with the aim of moving it towards a new model that provides good value for money and, at the same time, improves both the quality of the healthcare provided and makes it more accessible to the population that it is supposed to serve.

Across all levels, and throughout the world, healthcare is becoming more complex.^5^ In this complex environment, which is constantly and quickly changing, leadership means managing with an awareness of the nuances, constraints and limitations imposed.^6^ Management generally involves five tasks: managing self, managing systems or organizations, managing context, managing relationships and managing change.^7^

Good leadership and management are therefore all about providing direction and securing the commitment of partners and staff, facilitating change and delivering better healthcare services through the efficient, creative and responsible deployment of people and other resources.^8^ While leaders set the strategic vision and mobilize efforts towards its realization, good managers ensure effective organization and utilization of resources to achieve results and meet the aims.^9^

At present, lack of leadership and management capability is a constraint, especially at the operational levels of both the private and public health sectors.^9^

More than 25 years ago, Eitzen and Yetman^10^ reported research findings of great potential significance to theories of executive leadership. They found a curvilinear pattern between coach tenure and team performance in college basketball teams in the United States. The longer the coach's tenure was, the greater the team's success was. However, after a certain length of time (13 years on average), team performance declined steadily.^11^

More recently, strategic management scholars have emphasized the role of executive leadership in strategy formation and organization and system performance.^12^

In such a complex environment, it can be hypothesized that if the main leader, or the chief executive under any name, changes on a short-time basis, it is very hard to build a system that considers or is prepared for the medium and long terms, even if such public policies have been defined. In addition, it is a well known fact that the return on healthcare investments is normally seen only over the medium to long term, although returns are often difficult to measure, regardless of the time horizon.

## OBJECTIVES

The objective of this study was to describe and compare the average tenures of ministers of health in Brazil with those in countries with the highest Human Development Indices (HDI)^13^ over the course of the past 20 years. This study also looked at the relationship between ministers' tenures and a number of demographic, socioeconomic and healthcare and health indicators.

## MATERIAL AND METHODS

### Study design

This was a cross-sectional and exploratory study in which forty countries with the highest HDIs were initially selected, but only 23 were included. We searched the Ministry of Health websites for each country, listing the number of ministers and the length of their tenure over the past 20 years. When this information was not available on the website, a standard e-mail was sent to the contact address on the website. The e-mail message included a letter describing the purpose of the study and asked for the desired information, i.e. the number of ministers of health over the past 20 years and how long each one remained in office. If no response was received within two weeks, a second message was sent to the same e-mail address as well as to any alternate e-mail addresses identified at the Ministry of Health website. This procedure was repeated once more if no response was received within a further period of two weeks. All replies received within 45 days of the initial e-mail were considered for the study.

The number of Brazilian ministers of health and their tenures is available from the Brazilian Ministry of Health website.^14^

To gather the most recent socioeconomic, educational and healthcare indicators for the countries included in the study, we searched the World Health Organization (WHO)^15^ and World Bank^16^ websites. Descriptive statistics were used to characterize the data from each country. Pearson's correlation coefficient was used to correlate the number of ministers in each country over the past 20 years with that country's socioeconomic, educational and healthcare indicators. The Mann-Whitney test was used to compare ministers' tenures in Brazil and other countries.

## RESULTS

The Ministry of Health website of each country was surveyed and provided the required data for only eight countries (20%). Among the remaining 33 countries to whose ministries of health we sent an e-mail asking for data and describing the study purpose, 20 (61%) replied within 45 days. Fifteen countries (45%) answered with the required data and five countries (15%) stated that the data was not readily available or that they were unable or unwilling to provide it. Therefore, the required information was available for 22 of the 40 highest HDI countries (55%), plus Brazil, which is 70^th^ in the HDI ranking; all of these were included in our analysis.

Table 1 lists the countries included in this study, along with their HDI rank, the number of ministers of health over the past 20 years, and the mean number of months (with standard deviation, SD) for the ministers' tenure of each country. Switzerland, the 7^th^ ranked country on the HDI list, had the smallest number of ministers of health (three over the past 20 years). The Netherlands, the United States and Singapore, ranked 9^th^, 12^th^ and 25^th^, respectively, had a total of five ministers of health each over the course of the past 20 years. Among the top 40 HDI countries, Poland (ranked 37^th^) had 18 ministers of health over the past 20 years, the largest number in the study. Brazil had 17 ministers over the past 20 years. The mean (SD) tenure of Brazilian ministers of health is 15 (12) months, a period that is statistically significantly shorter than the mean tenure of 33 (18) months in the 22 other countries included in the study (P < 0.05).

**Table 1.** Median (with minimum-maximum, min-max), mean (with standard deviation, SD), number of months and number of ministers of health for each of the countries, in the last 20 years, ranked according to the Human Development Index (HDI), International Monetary Fund (IMF) and World Bank (WB) classifications of countries

HDI position	Country	Number of months median (min-max)	Number of months mean (SD)	Number of ministers
1	Iceland	26 (10−72)	34 (25)	8
2	Norway	36 (12–36)	27 (11)	8
3	Australia	36 (12–85)	42 (27)	8
4	Canada	24.5 (4–55)	26 (17)	11
5	Ireland	26 (1–58)	27 (22)	9
6	Sweden	23 (1–70)	25 (21)	11
7	Switzerland	96 (84–108)	96 (17)	3
9	Netherlands	26 (3–102)	39 (46)	5
11	Finland	30.5 (12–48)	30 (14)	9
12	United States of America	47.5 (37–96)	57 (26)	5
14	Denmark	21 (10–72)	26 (19)	10
15	Austria	31 (9–46)	30 (12)	9
16	United Kingdom	26.5 (17–44)	28 (9)	9
19	New Zealand	24 (8–71)	27 (19)	10
25	Singapore	42 (24–84)	48 (26)	5
26	South Korea	9 (0–21)	10 (5)	26
27	Slovenia	33 (6–61)	32 (20)	8
29	Portugal	35 (9–54)	34 (16)	9
32	Czech Republic	13 (1–29)	14 (10)	15
36	Hungary	18 (9–50)	20 (11)	13
37	Poland	13 (1–47)	13 (11)	18
40	Chile	32 (14–43)	30 (11)	10
	**Total (22 countries)**	**21 (0–108)**	**33 (18)**	**10 (5)**
70	Brazil	13 (0–47)	15 (12)	17
	**Total (23 countries)**	**20 (0–108)**	**32 (18)**	**10 (5)**
	**IMF (Advanced economies: 18 countries)**	23 (0–108)	28.6 (21.6)	9.05 (4.77)
	**IMF (Emerging and developing economies: 5 countries)**	16 (0–50)	17.2 (12.1)	14.6 (3.21)
	**WB (High-income economies: 20 countries)**	22 (0–108)	26.9 (20.7)	9.55 (4.77)
	**WB (Upper-middle-income economies: 3 countries)**	15.5 (0–47)	17.1 (13)	15 (4.36)

When the 23 countries were split into advanced economies (18 countries) and emerging and developing economies (five countries) using International Monetary Fund (IMF) criteria,^17^ the mean (SD) number of ministers of health in each group was 9 (5) and 15 (3), respectively.

Table 2 presents Pearson's correlation coefficient between the number of ministers of health over the past 20 years and a number of demographic and socioeconomic indicators. There was a statistically significant moderate positive correlation between the number of ministers over the period and a country's HDI rank. There were statistically significant moderate negative correlations between the number of ministers and the HDI index, *per capita* gross domestic product (GDP) (international purchasing power parity, PPP; United States dollars, US$), *per capita* gross national product (GNP) (international PPP; US$), percentage of the population with sustainable access to improved drinking water sources, percentage of the population with sustainable access to improved sanitation and registration coverage of deaths (%).

**Table 2.** Pearson correlation coefficients (PCC) between the mean number of ministers of health and each country's demographic and socioeconomic indicators (Brazil included)

	PCC	
	r	P
HDI position - 2007/2008 (n = 23)	0.522	0.011
HDI value - 2007/2008 (n = 23)	−0.517	0.011
Gross Domestic Product (GDP) (millions of US$) - 2007 (n = 23)	−0.188	0.390
GDP per capita (PPP US$) - 2005 (n = 23)	−0.598	0.003
Gross National Income (GNI) per capita (international PPP, US$) - 2006 (n = 23)	−0.632	0.001
Gini Index - 2007/2008 (n = 22)	0.024	0.916
Population, total (millions) - 2005 (n = 23)	0.065	0.769
Population annual growth rate (%) - 2006 (n = 23)	−0.230	0.290
Population in urban areas (%) - 2006 (n = 23)	−0.102	0.643
Population median age (years) - 2006 (n = 23)	−0.309	0.151
Population proportion over 60 (%) - 2006 (n = 23)	−0.313	0.146
Population with sustainable access to improved drinking water sources (%) (n = 19)	−0.693	0.001
Population with sustainable access to improved sanitation (%) - 2006 (n = 15)	−0.578	0.024
Adult literacy rate (%) - 2000/2004 (n = 5)	−0.572	0.313
Total fertility rate (per woman) - 2006 (n = 23)	−0.264	0.224
Registration coverage of deaths (%) (n = 23)	−0.455	0.029

HDI = Human Development Index; GDP = gross domestic product; PPP = purchasing power parity; US$ = United States dollars.

Table 3 shows Pearson's correlation coefficient between the number of ministers of health over the past 20 years and healthcare coverage, healthcare system resources, mortality and the burden of illness, and risk factor indicators for each country. There were statistically significant moderate negative correlations between the number of ministers and general government expenditure on health as a percentage of total government expenditure, per capita total expenditure on health at the average exchange rate (US$), per capita total expenditure on health (international PPP, US$), per capita government expenditure on health (international PPP, US$), nursing and midwifery personnel density, physician density (numbers per 10,000 population) and healthy life expectancy (HALE) at birth (years) for both genders. There were also statistically significant moderate positive correlations between the number of ministers and the age-standardized mortality rate for cancer (per 100,000 population), age-standardized mortality rate for cardiovascular diseases (per 100,000 population), age-standardized mortality rate for injuries (per 100,000 population), age-standardized mortality rate for non-communicable diseases (per 100,000 population), neonatal mortality rate (per 1,000 live births), and prevalence of tuberculosis (per 100,000 population).

**Table 3.** Pearson correlation coefficients (PCC) between the mean number of ministers of health and each country's health service coverage, health system resources, mortality and burden of illness indicators (Brazil included)

	PCC	
	R	P
**Health service coverage**		
Women who have had mammography (%) - 2002/2004 (n = 17)	−0.059	0.821
Women who have had Pap smear (%) - 2002/2004 (n = 16)	0.374	0.154
**Health system resources**		
General government expenditure on health as percentage of total expenditure on health - 2006 (n = 23)	−0.048	0.827
General government expenditure on health as percentage of total government expenditure - 2006 (n = 23)	−0.455	0.029
Per capita total expenditure on health at average exchange rate (US$) - 2006 (n = 23)	−0.581	0.004
Per capita government expenditure on health at average exchange rate (US$) - 2006 (n = 23)	−0.514	0.012
Per capita total expenditure on health (international PPP, US$) - 2006 (n = 23)	−0.567	0.005
Per capita government expenditure on health (international PPP, US$) - 2006 (n = 23)	−0.530	0.009
Private expenditure on health as percentage of total expenditure on health - 2006 (n = 23)	0.048	0.827
Out-of-pocket expenditure as percentage of private expenditure on health - 2006 (n = 23)	0.266	0.220
Total expenditure on health as percentage of gross domestic product - 2006 (n = 23)	−0.404	0.056
Hospital beds (per 10,000 population) (n = 22)	0.360	0.100
Number of nursing and midwifery personnel (n = 22)	−0.153	0.485
Number of physicians (n = 23)	−0.087	0.693
Nursing and midwifery personnel density (per 10,000 population) (n = 23)	−0.458	0.028
Ratio of nurses and midwives to physicians (n = 23)	−0.296	0.170
Physician density (per 10,000 population) (n = 23)	−0.419	0.047
**Mortality and burden of disease**		
Age-standardized mortality rate for cancer (per 100,000 population) - 2002 (n = 23)	0.542	0.008
Age-standardized mortality rate for cardiovascular diseases (per 100,000 population) - 2002 (n = 23)	0.508	0.013
Age-standardized mortality rate for injuries (per 100,000 population) - 2002	0.655	0.001
Age-standardized mortality rate for non-communicable diseases (per 100,000 population) - 2002	0.630	0.001
Neonatal mortality rate (per 1,000 live births) - 2004 (n = 23)	0.432	0.040
Infant mortality rate (IMR) - 2007 (n = 23)	0.324	0.132
Maternal mortality ratio (maternal deaths per 100,000 live births) - 2005 (n = 23)	0.323	0.133
Deaths among children under five years of age due to diarrheal diseases (%) - 2000 (n = 23)	0.302	0.162
Under-five mortality rate (probability of dying by age five years per 1000 live births) for both sexes - 2006 (n = 23)	0.319	0.138
Healthy life expectancy (HALE) at birth (years) for both sexes - 2003 (n = 23)	−0.560	0.005
Life expectancy at birth (years) for both sexes - 2006 (n = 23)	−0.506	0.014
Prevalence of tuberculosis (per 100,000 population) - 2006 (n = 23)	0.798	0.000

US$ = United States dollars, PPP = purchasing power parity.

## DISCUSSION

In this study, it was observed that in the countries with the highest HDI, the average minister of health tenure was 33 months. Interestingly, the average minister of health tenure over the past 20 years, in countries classified as advanced economies by the IMF, was almost twice as long (35 years) as for their counterparts in countries classified as emerging and developing economies (18 months).

Brazil, currently ranked 70^th^ according to the HDI, is a federation of 26 states and one Federal District. Over 5,500 municipalities enjoy federative status and political, administrative and financial autonomy. It has a national public health system, the so-called Unified Health System (Sistema Único de Saúde, SUS), which was formally created by the 1988 Federal Constitution. Nominally, the publicly funded SUS should provide full coverage to the entire Brazilian population, offering a complete range of services free of charge. It is the only service for over three-quarters of the population and the main provider for the poor. In fact, it is fraught with problems, mostly due to financing, management and structural causes, as well as governance failures, especially a lack of incentives and accountability that could be used to stimulate or enhance performance. The shortcomings of the national health service impact both healthcare management and delivery.^18^

The Brazilian Ministry of Health was created in 1953. Since that time, 41 ministers have been appointed. For the past 56 years the average tenure of Brazilian ministers of health has been 16 months. Only three ministers remained in office for longer than three years. Over the past 20 years, the average minister of health tenure in Brazil was statistically significantly lower than the average tenures observed in the 22 countries with the highest HDI that were included in this study (15 versus 33 months; P = 0.015).

It is interesting to note that the average tenure for soccer coaches among the 20 best teams in Brazil over this same 20 years period was around seven months.^19,20^ Thus, the average tenure for ministers of health in Brazil is twice that of soccer team coaches, but only half the tenure of their counterparts in countries with higher HDIs.

Brazil is currently a politically stable democracy. However, as in most developing nations, there is an element of political instability. Some parties are strong, yet not necessarily faithful to a given ideology. Senators, congressmen and elected and appointed executives have their own coalitions and interests. It is also fair to say that the ideological foundations of some parties or politicians are not rock solid, and decisions are often not made along party lines. The coalitions and power-sharing determine the best political arrangements for the time being. Political instability leads to uncertainty regarding the future of institutions and policymakers, which in turn affects the behavior of public and private agents.^21^

As Gordon and Rosen^22^ pointed out, newly appointed leaders do not function totally independently of their sponsors or of how those around them expect them to function.^11^ Political interests, personal agendas and different views of the world play an important role in defining the best arrangements for sharing and maintaining political power.

Changing an organization takes time; continuity of leadership to build a consistent internal system to drive desired behaviors and actions is crucial. Ministers of health are not always allowed to remain in office, in spite of satisfactory performance.

Some authors have described the tenure of CEOs as life cycles in which the executive has a steep learning curve when he or she first takes office, but then grows stale as he/she loses touch with the outside environment. Over recent years, a significant body of research has focused on the ways that top executives influence strategic choices and organizational performance.^23^ Interestingly, researchers have also found that organizations become reflections of their top executives.^23–25^

A few studies have examined how the impact of CEOs varies over their time in office. Hambrick and Fukutomi^11^ proposed that new CEOs begin with a knowledge deficit, but steadily learn about their jobs, organizations and environments. After some time, however, CEOs are thought to become insular and overly wedded to their early formulas, thereby resulting in an inverted U-shaped relationship between tenure and company performance. In line with this model, Miller and Shamsie,^26^ in a longitudinal study on the film industry, found that company performance increased for the first 8–10 years of a CEO's tenure and then began to fall.^23^

In 2002, Lucier et al.^27^ studied the CEOs of the world's 2,500 largest publicly traded companies who left office during 2001. The study included 231 CEOs. Their average tenure when they left in 2001 had been 7.3 years. The average tenure of CEOs in the healthcare industry was 9.1 years for CEOs who left office in 1995, 1998, 2000 and 2001. The average age at which executives became healthcare industry CEOs was 50.3 years for those leaving office in 1995, 1998, 2000 and 2001.^27^

More recently, Lucier et al.^28^ took a second look at the tenures of the CEOs of the world's 2,500 largest public companies, selected based on their market cap on January 1, 2006. Globally, the average CEO tenure had increased to 7.8 years, slightly higher than the average across the previous years studied. This average tenure was thought to provide enough time to implement an initial strategy, but also to allow for timely removal of poorly performing CEOs and those engaged in illegal and unethical behavior.^28^

Although these long average tenures pertain to a completely different environment, they allow us to think about and compare what happens in the healthcare system and private sector. In the UK, Taylor-Robinson et al.^29^ published a qualitative study that explored issues relating to the time horizons used in healthcare decision-making processes. This study showed that many public healthcare decision makers and policy makers felt that the timescales for decision-making were too short. Substantial systemic barriers to longer-term planning existed. Furthermore, it was felt that longer term planning was needed to address the wider determinants of health and to achieve changes at societal level. Three prominent `system’ issues were identified as important drivers of short-term thinking: the need to demonstrate an impact within the four-year political cycle; the requirement to `balance the books’ within the annual commissioning cycle; and the disruption caused by frequent reorganizations within the health service.^29^

As pointed out by one participant in the UK study, politicians probably have a shorter viewpoint because they are thinking about the next election. Also, many participants felt that the frequent reorganizations within the health service itself were particularly disruptive in terms of long-term planning. It was even stated there was no long-term perspective think tank.^29^

The present study also evaluated the relationship between the number of ministers of health over the past 20 years and some demographic, socioeconomic and health system and health indicators. Interestingly, we found a number of expected correlations, even considering that we were analyzing quite a homogeneous group of countries: with the exception of Brazil, the countries studied were those with the highest HDI and were mostly (18 of 23) classified as advanced economies by the IMF. In general, there was a statistically significant, moderate and inverse correlation between *per capita* or government expenditure and the number of ministers of health within the 20 year-period. The greater the *per capita* expenditure was, the lower the number of ministers was. It is also interesting to note that there was a relationship between the number of ministers and the mortality and burden of disease indicators. The higher the mortality rate for several conditions was, the lower the number of ministers within the last 20 year-period was (most of the correlations were moderate and statistically significant).

This study has some limitations, since it merely uses average tenures within a certain period of time and correlates them with the country's demographic, socioeconomic, healthcare system and health indicators. Although tempting, it is not possible to establish any causal relationship. We can certainly develop hypotheses to address the issue of whether or not managerial tenure "causes" organizational and systemic outcomes, but further studies are required to document and assess the rationale behind such a hypothesis.

It is important to emphasize that leadership and management are complex concepts that are relevant to many different parts of the healthcare system, including the private and public sectors, healthcare facilities, district health offices and central ministries, as well as the support systems relating to drugs, finance and information. Leadership and management are also human resource issues: specifically, the skills and motivation that managers and leaders need to work throughout a health system.^9^

Furthermore, individual political behavior and political outcomes in any society are constrained by its political institutions.^30^ Political institutions provide the structure for collective decision-making and define the context for resource redistribution and public good provision by governments.^31^ Long-term policymaking involves current decisions with distant consequences. Any choice that society makes today has consequences for other choices that public and private players and subsequent societies can and will make in the future.^32^

Care, in most countries, continues to be fragmented, unsafe and inefficient. As stated by Corrigan and McNeill, achieving high levels of performance requires organizational capacity, including information technology and specialized expertise that are not present in most settings. Therefore, a comprehensive policy agenda is needed to encourage growth in organizational capabilities, including national priorities and goals, performance measurement and reporting, payment or compensation reform, community leadership, information technology and public education.^33^

In conclusion, the Minister of Health is a key decision-maker in healthcare systems and has the potential to highly influence policy-making. The Minister of Health is usually responsible for providing overall direction and leadership for the system, focusing on planning and on guiding resources to bring value to the healthcare system. Constant change in an important office such as that of the Minister of Health is, in and of itself, a threat and a sign of poor sustainability and continuity of the decision-making process and long-term healthcare policies. At the same time, it may be a consequence of a short-term view of the political environment, in which decision-making and policy-making are in a sense restricted to the elected mandate. It also has the potential, over the short term, to solve only the problems and satisfy only the needs and interests of specific groups, which may be another threat to the establishment of a system that should be committed to the community as a whole.

While the challenges that developed and developing nations face are great, the opportunities for improvement of their healthcare systems are even greater. Significant resources are currently wasted, in part because of a lack of guided and justified decisions that take into consideration not only the infrastructure in place, but also the adequacy of the time frame within which they are based. In spite of the short-term requirements, there is an urgent need to think and plan healthcare systems from a long-term perspective. A more efficient healthcare system focused on long-term policies and transparent and justified decisions must be the goal.

## CONCLUSION

On average, ministers of health have extremely short tenures. There is an urgent need to think and plan healthcare systems from a long-term perspective.
